# Transposition Behavior Revealed by High-Resolution Description of *Pseudomonas Aeruginosa* Saltovirus Integration Sites

**DOI:** 10.3390/v10050245

**Published:** 2018-05-07

**Authors:** Gilles Vergnaud, Cédric Midoux, Yann Blouin, Maria Bourkaltseva, Victor Krylov, Christine Pourcel

**Affiliations:** 1 Institute for Integrative Biology of the Cell (I2BC), CEA, CNRS, Univ. Paris-Sud, Université Paris-Saclay, 91198 Gif-sur-Yvette CEDEX, France; cmidoux@gmail.com (C.M.); yann.blouin@gmail.com (Y.B.); christine.pourcel@u-psud.fr (C.P.); 2 I. I. Mechnikov Research Institute for Vaccines & Sera, Moscow 105064, Russia; mariabour@mail.ru (M.B.); krylov.mech.inst@mail.ru (V.K.)

**Keywords:** transposable phages, transposon integration, hotspots, deep sequencing, chromosomal domain, supercoiling, imaging

## Abstract

Transposable phages, also called saltoviruses, of which the *Escherichia coli* phage Mu is the reference, are temperate phages that multiply their genome through replicative transposition at multiple sites in their host chromosome. The viral genome is packaged together with host DNA at both ends. In the present work, genome sequencing of three *Pseudomonas aeruginosa* transposable phages, HW12, 2P1, and Ab30, incidentally gave us access to the location of thousands of replicative integration sites and revealed the existence of a variable number of hotspots. Taking advantage of deep sequencing, we then designed an experiment to study 13,000,000 transposon integration sites of bacteriophage Ab30. The investigation revealed the presence of 42 transposition hotspots adjacent to bacterial interspersed mosaic elements (BIME) accounting for 5% of all transposition sites. The rest of the sites appeared widely distributed with the exception of coldspots associated with low G-C content segments, including the putative O-antigen biosynthesis cluster. Surprisingly, 0.4% of the transposition events occurred in a copy of the phage genome itself, indicating that the previously described immunity against such events is slightly leaky. This observation allowed drawing an image of the phage chromosome supercoiling into four loops.

## 1. Introduction

Transposable phages, which include the *Escherichia coli* Mu and similar Mu-like phages, are temperate phages that can persist in their host as a prophage [1]. The infection is initiated by the attachment of the virion fibers to the host cell surface lipopolysaccharides. The phage DNA is injected together with the MuN protein present in the virion, which then binds to the ends of the viral genome and converts it into a noncovalently closed circle prior to integration into the host genome. During the lytic cycle, the phage genome is multiplied by replicative transposition at 50–200 sites per bacterial genome with no specificity for a particular insertion site, and packaged together with host DNA flanking the phage genome insertion site [2]. For instance, the linear 37 kb Mu phage double-strand DNA genome is packaged with 50–150 base-pairs of DNA fragments of the *E. coli* host genome on the left side from which packaging is initiated [3] and a few hundred base-pairs up to 2–3 kb on the right side resulting from full-head packaging [4]. Two phage-coded proteins, MuA and MuB, are essential for Mu transposition (reviewed in References [5,6]). MuA, a DDE recombinase, binds to the phage attachment sites (attL and attR) and to the phage transposition enhancer region whereas MuB, an AAA+ ATPase binds nonspecifically to the host chromosome and is therefore involved in the selection of insertion sites [6,7,8,9]. MuB assembles into a polymer that samples the chromosome, by assembly and disassembly involving MuA, until an appropriate site is located for the insertion of a bacteriophage genome [10,11].

Whereas numerous reports have investigated the mechanism of transposition of the Mu genome both in vivo and in vitro (reviewed in References [6,7,8,12]), relatively few have addressed the nature and diversity of the transposon integration sites during replicative transposition [13,14,15]. Manna et al. reported that genes with a high frequency of the triplet CGG were preferred sites for Mu transposition [16]. Haapa-Paananen et al. identified a MuA encoded preference for the 5-bp target recognition site, 5′-CYSRG-3′ [17]. A mechanism called target immunity, involving the protein MuB, prevents insertions in copies of the Mu genome [18,19,20]. The formation of MuB helical filaments in the process of transposition may protect the Mu DNA from self-insertion [21]. In the absence of such a mechanism, virion production would be significantly impaired since the viral genome (37 kb) integrating into the *E. coli* K-12 chromosome (4.6 Mb) during replication may eventually represents more than 50% of the total DNA.

Transposable phages are present in different bacterial species [12]. Whereas Mu is a myovirus, the known examples of the *Pseudomonas aeruginosa* transposable phages are siphoviruses (long, non-contractile tail). In most other regards, their lifestyle is very similar to Mu. Initially based on DNA-DNA homology and more recently on whole genome comparisons, they have been distributed into two groups related to phages D3112 and B3, respectively [22,23,24,25]. The genome organization of the D3112 group is Mu-like, with all genes transcribed in the same direction except for the left-most one, the lysogeny repressor. The B3-like genome organization differs by an inversion of the early genes operon [24,26]. Additional members of the D3112 group have been described, often as prophages within *P. aeruginosa* whole genome sequences, and their genome was compared showing the existence of core and accessory genes and of regions of high heterogeneity [23,27]. Mu and B3-like phages code for different transposition proteins [26,28].

A very low frequency of integration events causing auxotrophic mutations was observed among D3112 lysogens in sharp contrast with B3-like or Mu lysogens, suggesting that the two groups display different insertion site specificities during lysogeny [29,30]. Mini-D3112 transposable elements have been used for genetic analysis of *P. aeruginosa*, allowing an investigation of the prophage insertion sites, and showing that none of the 1000 analyzed sites were associated with auxotrophy [30].

Until recently, the number of investigated insertion targets was dependent on mutations and was too low to draw conclusions on their precise distribution and to investigate if different transposable phages and host combinations might behave differently. In the present work, using the Ab30 phage from the D3112 group, we take advantage of deep sequencing to investigate the diversity and distribution of the transposon integration sites during replication at an unprecedented resolution. We demonstrate the presence of major hotspots superposed to a globally random distribution of transposition events except for AT rich regions which are coldspots. We also precisely measure the efficiency of the target immunity and use the observed distribution to propose that the phage domain is organized in four loops. We compare the transposition sites distribution in two other phages from the D3112 group, phages 2P1 and HW12, to show that two patterns of transposition are observed.

## 2. Materials and Methods

### 2.1. Transposable Phages Origin

Phage Ab30 was isolated from sewage water in Abidjan (Côte d’Ivoire) [31], whereas phage 2P1, producing turbid plaques, was isolated from a sewage water sample in Orsay (France) and phage HW12 was isolated in Moscow (Russia) from a *P. aeruginosa* clinical isolate. The three siphoviruses show a restricted host spectrum as is usual for temperate phages and could not all be grown on the same *P. aeruginosa* strain. The main metadata are listed in Table 1.

### 2.2. DNA Preparation

The phages were amplified on fresh Luria Broth (LB) agar plates at a multiplicity of infection (MOI) of one phage for 1000 bacteria. An overnight culture of bacteria in LB was concentrated 10 times in saline magnesium (SM) phage buffer (SM is 50 mM Tris-HCl pH 7.5; 100 mM NaCl, 8.1 mM MgSO4, 0.01% gelatin). The phages were added and after 15 minutes at room temperature, the mixture was poured with 4 mL of soft agar onto a round plate. After complete bacterial lysis (≈8 h), 5 mL of SM phage buffer and a drop of chloroform were added to the recovered soft agar containing phage particles.

After centrifugation at 1000 *g*, the Ab30 or 2P1 phages in the supernatant were purified by polyethylene glycol (PEG) precipitation, followed by 0.22-μm-filtration and ultracentrifugation as previously described [31]. Phage HW12 was purified by a cesium chloride gradient instead of PEG precipitation. DNaseI was added to the phages at a concentration of 50 µg/mL before incubating for one hour at 37 °C prior to phage DNA purification.

The phage and bacterial DNA were purified using phenol/chloroform as described in Reference [32]. The purified DNA was suspended in TE buffer (10 mM Tris-HCl, pH 7.5, 1 mM EDTA). The quality and concentration of the DNA were measured using an ND-1000 Spectrophotometer (NanoDrop^®^, Labtech, Palaiseau, Paris, France) and a Qubit fluorimeter (Thermo Fisher Scientific, Villebon-sur-Yvette, France).

### 2.3. Enrichment of the Right End of Phage Ab30 to Produce a Library of Ab30 Transposition Sites

The purified DNA from Ab30 virions grown on the *P. aeruginosa* strain PA14 was digested with the restriction enzyme *Nsi*I that rarely cuts the bacterial host genome (once every fifteen kilobases, on average). *Nsi*I was chosen because the Ab30 phage genome contains only two recognition sites for this enzyme. The first is located at position 6170 bp and the second is located at position 37,024 bp, very close to the phage right end at position 37,205 bp. Digestion produces a left-end fragment of 6170 bp plus 31–36 bp attached bacterial host DNA, one large internal fragment of 30,854 bp, and the right-end fragment of 181 bp plus bacterial host DNA of variable length (Figure S1). The smear corresponding to the right-end fragments was separated into two fractions, one from 170 bp–900 bp (fraction A) and the second from 900 bp–2500 bp (fraction B). The DNA recovered from fraction A could be directly used for TruSeq library preparation and Illumina sequencing whereas the DNA from fraction B was mechanically broken to produce fragments of mean size 500 bp.

### 2.4. Sequencing and Sequence Data Management

Deep sequencing was performed by the High Throughput Sequencing platform of I2BC (HTS, I2BC, Gif-sur-Yvette, Paris, France) using MiSeq System (Illumina Solutions Center Paris, Evry, France). Two million 250 bp reads were produced for the sequencing of phage HW12. Three million 250 bp or 300 bp reads were produced for the sequencing of phage 2P1. One million 100 bp reads was initially produced for the sequencing of phage Ab30. The reads datasets were deposited in bioproject PRJEB12851, reads accession numbers are indicated in Table 1. In addition we sequenced the clinical PcyII-10 *P. aeruginosa* strain which was used to recover and cultivate phage 2P1, and resequenced the PA14 reference strain used in our laboratory (PA14Or) to grow phage Ab30. These genomes sequences used solely as controls can be recovered under accession number LT673656.1 and LT608330.1 respectively.

The Ab30-derived A and B libraries were sequenced using two Illumina MiSeq runs producing 250 bp paired reads. Twenty-two million and fourteen million pairs of reads were produced from Ab30 transposition sites libraries A and B, respectively. The full library A and library B dataset correspond to run accession ERR1596854 and ERR1596855, sample accession SAMEA4393803 and SAMEA4393804, in bioproject PRJEB12851. All sequence data files quoted previously can be accessed at http://www.ebi.ac.uk/ena/data/view/<ACCESSION NUMBERS>.

Sequence data were processed using Geneious R11 (Biomatters Ltd, Auckland, New Zealand). The reads were mapped on the phage genomes using the Geneious mapper with default parameters. Reads overlapping the phage ends were trimmed of the phage sequence.

An additional filter was applied to the reads from the A and B transposition sites libraries prior to trimming in order to eliminate potential artefacts. Only reads starting precisely from the *Nsi*I restriction site of the 181 bp right-end phage sequence and containing at least 12 bp of the bacterial host DNA were subsequently conserved. Eventually, 10,503,241 and 2,880,145 reads from the A and B libraries respectively satisfied these strict selection criteria (“valid reads”). The 181 bp right-end phage sequence was trimmed using Geneious.

In order to draw plots of transposition sites, the first non-trimmed base was annotated as “start”. All trimmed and start-annotated reads were mapped on the appropriate reference genome using the Geneious mapping tool with default parameters except for the maximum number of gaps per reads (set to 3%), maximum mismatches per read (2%) and minimum overlap identity (95%). After mapping, the “start” annotations from the reads were transferred to the reference genome. The information associated with the transferred annotations was then exported from Geneious as a text file containing mapped reads Id, mapping orientation, and position of the “start” in the reference genome. This data file was used as input by plot drawing python scripts deposited at https://github.com/midoux/GPMS/.

The making of TruSeq Illumina sequencing libraries involves a few PCR cycles which may produce chimeras between initially independent DNA molecules. Such chimeras may subsequently be confused with phage-host junctions when they occur precisely at the phage chromosome end. We evaluated the proportion of such artefacts among sequencing reads by mapping a phage read archive on truncated phage genomes. For this purpose we mapped the 2P1 sequence reads dataset (Table 1) on four 2P1 phage genome sequences shortened of 1 kb, 10 kb, 20 kb and 30 kb at their left end. We recovered the pseudo-junctions overlapping the artificial phage chromosome ends and mapped them on the *P. aeruginosa* PcyII-10 genome. From these four ends, we recovered a total of 19 candidate junctions which represent the maximum number of artefacts (average 5 events per end). In contrast more than 19,000 potential junctions are recovered when mapping on the bona fide 2P1 phage left-end.

### 2.5. Statistical Analysis of the Distribution of Transposition Events

The expected number of coincident integration events under a random distribution model was calculated as described in Reference [33]. If transposition events are randomly distributed along the genome, the expected number of coincident events depends on the number T of transposition events characterized, and R the ratio of T to the genome size. If T is 25,000 and genome size is 6.3 Mb, R is 1/250. R being lower than 0.1, the expected number of double coincidences E2 is closely approximated by E2=TR/2, i.e., 50 positions with two coincident integrations among 25,000 events. The expected values for higher coincidences is E3 = ln(1/(1 − R))E2/3 and Ei + 1 = ln(1/(1 − R))Ei/(i + 1).

## 3. Results

### 3.1. The Different Transposition Site Distributions Observed in the Three Phages

The genome sequence of phage Ab30 was previously determined as part of a study of bacteriophages from Abidjan, using a total of one million reads providing an average phage sequencing coverage of 1000X [31]. In the present work, we characterized the genome of two new phages, 2P1 and HW12, with three and two millions sequencing reads (phage sequencing coverage 10,000X and 12,000X, respectively). Phages Ab30, 2P1, and HW12 shared the Mu-like genetic organization [26] and their genome ends appeared to be flanked by variable ends (VEs) originating from the bacterial host. Additional similar phages were identified by a blast search against the Genbank non-redundant nucleotide collection. Figure S2 shows the result of a clustering analysis of the *P. aeruginosa* saltoviruses genome sequences. All three phages belong to the D3112 group, Ab30 clustered in the DMS3 subgroup, 2P1 in the D3112 subgroup, whereas HW12 was more distantly related. The genome of all the phages in the D3112 group starts with TGT and finishes with ACA in agreement with previous observations [23].

We recovered 2500, 19,300, and 25,150 sequences potentially corresponding to host DNA attached to the left-end of the Ab30, 2P1, and HW12 genomes, respectively. These values are higher than the average sequencing coverage as expected when sequencing a linear chromosome and indirectly confirms that these sequencing reads originate from the ends of the DNA molecule as present in the virions. Such chromosome ends are expected to be overrepresented in an Illumina TruSeq sequencing library produced by mechanical shearing of the DNA [31,32,34]. The size of the left-end host DNA fragments was predominantly comprised between 31 bp and 36 bp as previously observed in RcapMu [14] and in agreement with early observations in the D3112 group [25].

**Figure 1:**
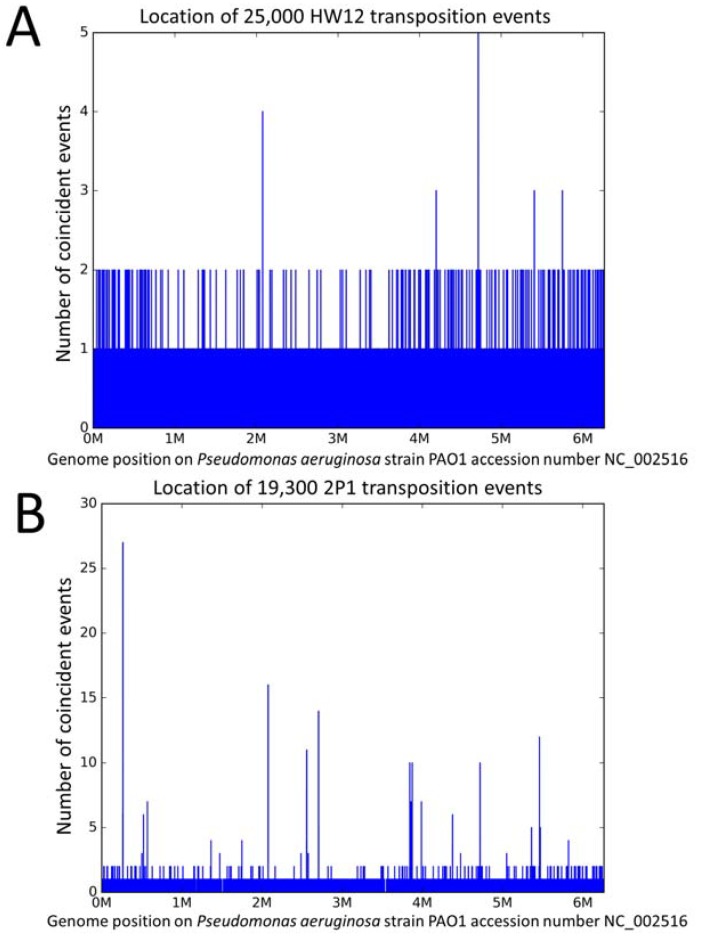
Distribution of transposition sites on *Pseudomonas aeruginosa* at base-pair resolution. Panel (**A**) The distribution of 25,000 HW12 transposition sites. A maximum of five coincident insertions (one in the reverse orientation and four in the forward orientation) is observed at position 4,720,184. Panel (**B**) The distribution of 19,300 2P1 transposition events. Eight peaks above ten coincident insertions are observed. The tentative HW12 hotspot is one of them. The highest peak corresponds to 27 coincident insertions at positions 265,012–265,013.

We then mapped the host DNA fragments on the *P. aeruginosa* genome in order to locate the transposition sites. Figure 1A shows the location of the 25,150 phage HW12 left-end host fragments mapped on PAO1.

The amount of forward/reverse HW12 integrations along the PAO1 genome was 12,501 and 12,649, respectively, with a majority of unique transposition locations. One hundred and sixty-eight positions were occupied by two transposition events. If the distribution was random, 50 occurrences of two and 0.066 events of three coincident insertions in the whole genome would be expected (Table S1). A maximum of five insertions was observed at the PAO1 position 4,720,184. One insertion event occurred in the reverse orientation whereas four occurred in the forward orientation. Furthermore, it is noteworthy that two among the five peaks with three coincident insertions were located immediately adjacent at positions 4,720,179 and 4,720,187. The 34 bp PAO1 segment 4,720,162–4,720,195 with a total of 22 transposition events represents a tentative hotspot (0.09% of all events, i.e., a 150 fold excess).

The distribution of transposition events in phage 2P1 was strikingly different. Figure 1B shows the location of the 19,300 left-end host DNA fragments from phage 2P1 mapped on the *P. aeruginosa* PAO1 reference genome (See also Figure S3, mapping of the sites on the host strain genome *P. aeruginosa* PcyII-10). Two hundred and sixteen positions with two coincident insertion events were observed, slightly more than the 168 observed with HW12. However, remarkable peaks were detected. For instance, 27 insertions (13 forward and 14 reverse insertions) coincided at position 265,013. Comparison with the expected values under a random distribution model is shown in Table S1. A total of 142 transposition events clustered within positions 265,009–265,025 (that is, 0.75% of all events clustered in a 17 bp segment (a more than 3000 fold excess)). The tentative HW12 hotspot at position 4,720,184 in Figure 1A was also present. Forty-eight insertion events occurred within the 13 bp segment 4,720,179–4,720,191.

We similarly mapped the 2500 left-end host fragments from Ab30 sequencing data (Table 1). Interestingly, a maximum of nine insertions was observed at the PAO1 position 4,720,182, i.e., within the 2P1 and the tentative HW12 hotspot. In spite of the relatively low number of available Ab30 events in this initial dataset, additional hotspots could be detected. For instance 22 events occurred in the 13 bp segment 265,009–265,021, representing a 1700 fold excess. This position coincides with the strongest 2P1 hotspot in Figure 1B.

In conclusion, the distribution of the HW12 transposition events in its PAO1 host appeared to be mostly random, with however a slight but significant excess of coincident transposition sites at one or two positions, whereas the distribution of the 2P1 and Ab30 transposition sites displayed several major hotspots.

### 3.2. Distribution of the Ab30 Transposition Sites

**Figure 2:**
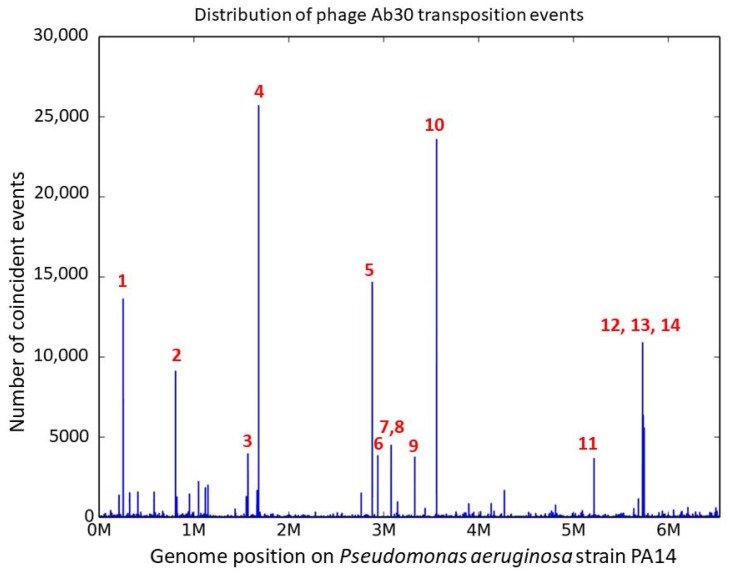
The phage Ab30 major transposition hotspots. The distribution of the 13 million Ab30 transposition events is plotted along the PA14 bacterial host genome at a one base-pair resolution. On the vertical axis, the number of transposition events at a particular position is shown. The 14 sites with at least one position hosting more than three thousand insertions are numbered.

We decided to focus on phage Ab30 and in order to get access to a larger number of transposition sites, we designed an experiment to preferentially sequence the bacterial DNA attached to one end of the phage genome. Following the described enrichment procedure, we obtained 13,383,386 reads containing the right-end junction of a phage transposition event and mapped them on the PA14 host genome revealing a number of transposition hotspots (Figure 2).

**Figure 3:**
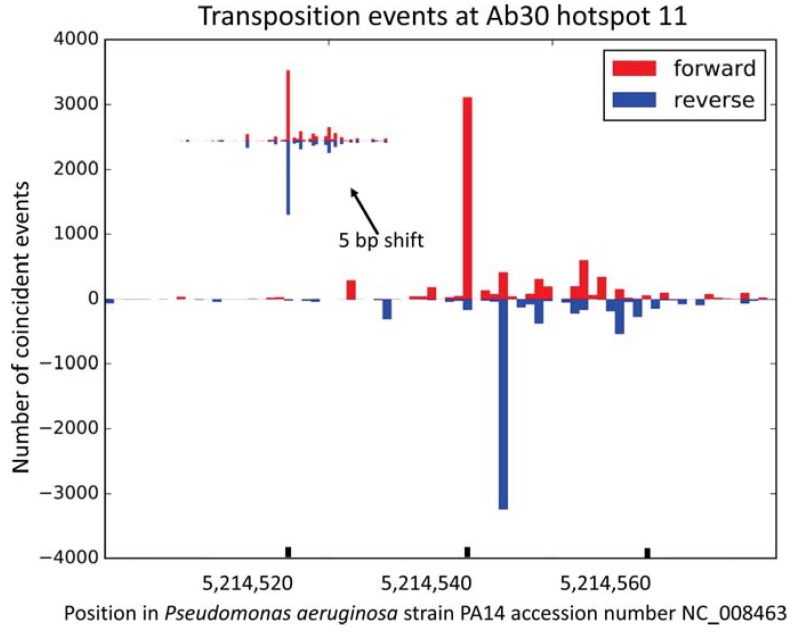
A focus on Ab30 hotspot 11. The position of the transposition events is shown at a one base-pair resolution. Insertions in the forward or reverse orientations are presented in the red or blue colors, respectively. The small inset is obtained by a five base-pairs shift of the upper part of the graph. The almost perfect symmetry illustrates the absence of bias in transposition orientation.

Whereas an average insertion frequency of two per nucleotide would be expected on the 6.5 Mbp bacterial genome, about 30 nucleotide positions were targets for 500 insertions or more. Fourteen hotspots numbered 1–14 in Figure 2 showed peaks of more than 3000 coincident insertions at a one nucleotide resolution. Half of the fourteen hotspots were localized inside coding sequences and the other half were intergenic (Table 2). Figure 3 presents a focus on hotspot 11 illustrating the absence of bias in the insertion orientation.

### 3.3. Presence of a Consensus Sequence Adjacent to the Hotspots

**Figure 4:**
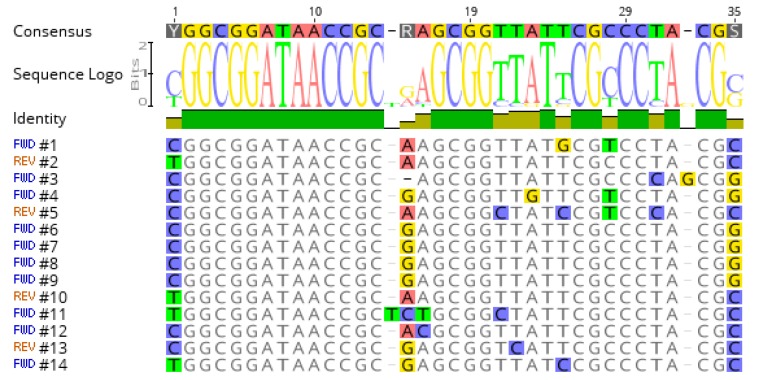
Definition of a highly conserved consensus. The conserved sequences adjacent to each of the fourteen hotspots are shown with the 35 bp consensus sequence on top.

We then searched for the basis of the preferred insertion at a limited number of hotspots by aligning the 280 bp flanking the fourteen highest peaks using MAFFT [37]. This showed a good alignment over a 35 bp sequences at 55–61 bp on one side of the hotspot (Figure S4), and the consensus sequence 5′-YGGCGGATAACCGCRAGCGGTTATTCGCCCTACGS-3′ could be deduced from the comparison (Figure 4). This sequence is 65% GC, identical to the average GC content of the *P. aeruginosa* genome. It resembles the palindromic unit or “repetitive extra palindromic” (REP) sequences of the “bacterial interspersed mosaic elements“(BIME) of *E. coli* [38].

In *P. aeruginosa*, some BIME elements are tandemly repeated and polymorphic. Accordingly, four among the 14 major hotspots corresponded to previously described Variable Number of Tandem Repeats (VNTRs), namely hotspot 1 (ms211), 6 (ms214), 12 (ms223), and 14 (ms224). This observation suggested that the succession of sites augments the attractiveness for phage insertion. Three other VNTRs (ms142, ms215, and ms222) were associated with minor hotspots (Table 2, Tables S2 and S3) [36].

The hotspot consensus sequence was searched for in the total PA14 genome, with a three mismatches tolerance, allowing the identification of 42 occurrences (Table S3). The reads were aligned against these regions showing that about 5% of the insertions occurred at these positions. In total, these 42 sites represented less than one kilobase within the 6500 kb bacterial genome. There is, therefore, a more than 300 fold bias towards the insertion at one of these positions.

Apart from the 5% reads mapping to the main hotspots, the insertions appeared to be randomly distributed. In order to illustrate the difference of transposition sites distribution in Ab30 as compared to HW12, and similarity with phage 2P1, a random subset of 25,000 Ab30 transposition sites were mapped on the PAO1 genome (Figure S5). Table S1 provides a more detailed counting of sites with multiple coincident insertions.

### 3.4. Coldspots for the Transposition Events Corresponding to Low GC Content Regions

The density of transposition sites characterized here allowed not only the detection of hotspots, but also of a number of remarkable coldspots. Sixteen regions of at least one kilobase in length showed a density of less than ten Ab30 transposition events per kilobase compared to the global average of 1300–1800 events per kilobase observed across the genome outside of the hotspots. These coldspots corresponded to the low (less than 50%) GC-content regions as compared to the 65% GC average in *P. aeruginosa* (Figure S6). For instance, the 1530 bp segment 4,399,680–4,401,210 inside the gene for Acyl-homoserine acylase *quiP*, with a 42% GC-content, was devoid of transposition events. Only 83 transposition events mapped to the largest 13 kb long coldspot (PA14 accession number NC_008463 segment 2,027,000–2,040,000). This 46% GC region delimited at one end by the O-antigen chain length regulator gene *wzz* and at the other end by *wbpM* corresponded precisely to the O-antigen biosynthetic locus [39]. The three 16S–23S operons in the PA14 genome, covering 15 kilobases with a 52% GC content, showed a slightly higher transposition density of 30 events per kilobase.

### 3.5. Insertion of Ab30 into the Already Inserted Copies of Ab30

**Figure 5:**
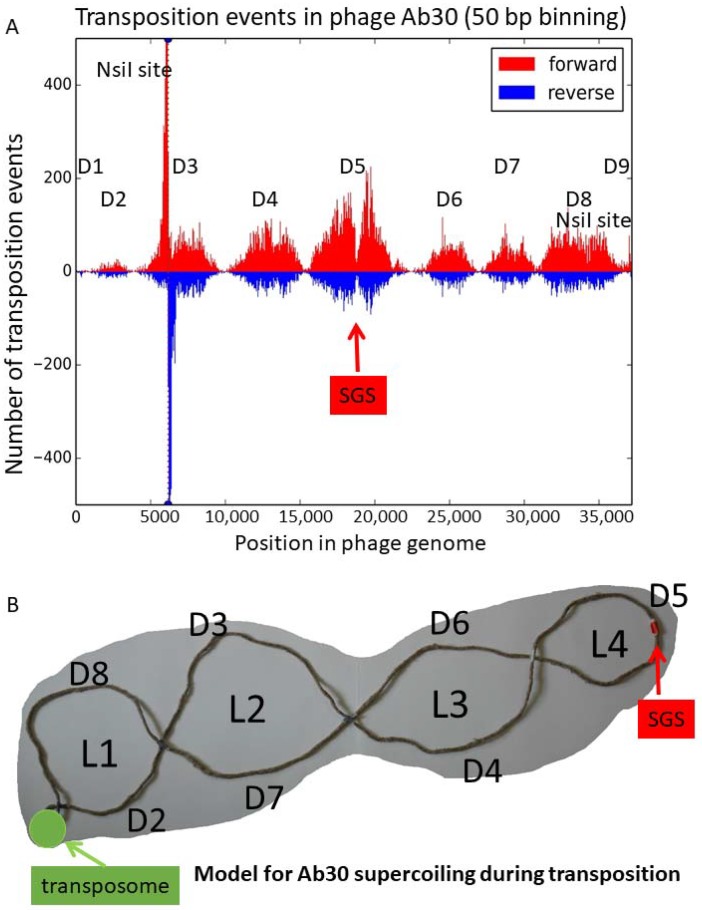
The discovery of a pattern of transposition events along the Ab30 genome. The position along the phage Ab30 genome of transposition events which occurred within the phage is plotted. (**A**) The number of reads in 50 bp bins is shown on the axis. The orientation of the insertion is depicted with colors. The position of the two *Nsi*I sites present in the Ab30 genome is indicated. The *Nsi*I site at position 6170 creates an artifact hotspot. Seven main peaks are observed defining nine domains numbered accordingly. (**B**) Proposed topology of the phage genome. The pattern of transposition events along the phage genome presented in panel (**A**) defining domains D1 to D9 suggested the existence of four loops (L1–L4) including four fixed crossing points inaccessible to transposition. The locations of the synaptonemal complex (transposome) and predicted strong gyrase site (SGS) are indicated.

Very interestingly 53,800 transposition sites corresponded to the Ab30 sequence itself, indicating that 0.4% of the transposition events escaped target immunity and occurred inside a previously transposed copy of the viral genome. A remarkable distribution of reads was observed with the existence of nine domains of unequal size and density of the integration events (numbered D1–D9 in Figure 5A).

The domains D1 and D9 with sizes of 900 and 700 bp, respectively, corresponded to the phage genome ends. A very small peak was visible within the first few hundred base-pairs in domain D1. The end of the domain D1 appeared to correspond to the location of the transposition enhancer region E in the phage Mu (reviewed in [9]). In the D9 domain, beyond position 36,500, the situation was unclear as most of the minor peak was probably an artifact due to the presence of the second *Nsi*I site, located 180 bp before the phage genome end. Domain D2 started approximately 900 bp from the phage left-end and was the next smallest in size (2900 bp) and transposition site density (958 transposition events, a density of 330 events per kilobase). The *Nsi*I site at position 6170 within the 6100 bp domain D3 explained the presence of the two flanking and symmetrical sharp peaks. The peaks expanded for approximately one kilobase on both sides. Apart from this artifact, domains D3 and D4 were similar in size (6100 and 5300 bp, respectively) and transposition site density. The central domain, D5, was the largest in size (7200 bp) and in the number of sites (9594 transposition events, with a density of 1332 events per kilobase). Domains D6 and D7 were also similar in size (4500 and 3800 bp, respectively) and in site density (densities of 620 and 709 events per kilobase, respectively). The domain D8 covered 5800 bp and showed the second highest density of transposition events: 1049 per kilobase.

The middle of the largest domain D5 showed a sharp decrease in transposition events. Figure S7 illustrates the location of the phage insertions at a one base resolution in this region. The 37 bp intergenic segment from position 18,736–18,772 in the Ab30 genome was devoid of phage insertions. Previous works on phage Mu identified a “Strong Gyrase Site” (SGS) located near the middle of the phage genome and showed that binding of the gyrase protected a core region of 30–50 bp within the SGS [40,41]. It is possible that the transposition-free segment in the middle of phage Ab30 marks the position of the SGS, 150 nucleotides to the right of the exact middle of the phage genome. This hypothesis implies that the transposition events occur in phage genome copies topologically organized by the gyrase. The pattern of insertions shown in Figure 5A likely reflects the supercoiling of the phage genome [42] and the accessibility of the different loops. In type phage Mu, the sequence to the left of the left-most CDS—the C-repressor—contains the *attL* (L) site, whereas the sequence to the right of the right-most CDS contains the *attR* (R) site. The AT-rich intergenic segment to the right of the C-repressor contains the transposition enhancer (E) site. In the transposome, MuA binds and maintains together the L, R, and E sites ([43]; reviewed in References [1,9]). We propose in Figure 5B a graphical representation of the supercoiling of the Ab30 genome during the transposition stage. In this model, the phage genome is organized into four loops in addition to the transposome located at the basis of the phage domain. The first and second loops are very asymmetrical. In loop L1, the 2900 bp domain D2 faces the 5800 bp domain D8. D8, which represents two-thirds of the loop, attracts 86% of the transposition events. In loop L2, the 6100 bp domain D3 is facing the 3800 bp domain D7. In loop L3, the 5300 bp D4 is facing the 4500 bp D6. Loop L4 is composed of the 7200 bp domain D5 which is the most symmetrical and the most attractive in terms of transposition events—with 9600 occurrences. This corresponds to the average 1300 transposition events per kilobase compared to the 800 events per kilobase in loops L1 and L2. The SGS would be located at the apex of L4.

## 4. Discussion

Deep sequencing of three *P. aeruginosa* transposable phage genomes (HW12, 2P1, Ab30) was used to investigate tens of thousands up to 13 million phage-host DNA junctions, the trace of replicative transposition sites. We observed two distinct distribution patterns of transposition site suggesting, for the first time, that these phages display different transposition behaviors. Whereas one pattern is mostly random, the second shows a few very high hotspots located in the vicinity of BIME structures, widespread among bacteria [44,45,46]. Their capacity to form hairpins might be attractive to some transposable phages. Alternatively, the bacterial host may be responsible for the control of these hotspots, as the three phages were grown on three different strains due to their limited host range. It is interesting to note that some IS elements show a preference for insertion in similar repetitive extragenic palindromic sequences [35,47,48]. In *P. aeruginosa*, ISPa11 inserts frequently into the intergenic VNTRs recalled in the present study [35]. This could be related to the secondary structure of the target site and/or to the characteristics of the transposase. In *E. coli*, some BIMEs are target for DNA gyrase which may have consequences for DNA topology [49,50]. It was shown that in Eukaryotes, transposon and retroviral insertions were frequent in regions of DNA flexibility [51]. The work of Ton-Hoang et al. analyzed a new clade of Y1 transposases associated with REP/BIME in *E. coli* and responsible for the dispersion of these structures into the bacterial genome [52]. Y1 transposases are widespread and found in some phages. It was shown that the histone-like nucleoid structuring protein (H-NS) which preferentially binds bent and deformed sequences [53] regulates the transposition of Tn5 and Tn10 by interacting with the transposition machinery [54]. It is possible that H-NS also interact with BIMEs, facilitating the insertion of Ab30 at these specific locations. IHF (Integration Host Factor), another nucleoid binding protein was shown to bind BIMEs sequences and induce topological constraints targeted by DNA gyrase [50].

The high resolution mapping of transposition sites achieved for phage Ab30 revealed additional patterns of distribution within a given host. DNA stretches with a low GC ratio (that is, below 50% in the *P. aeruginosa* genome with a 65% average GC content) are coldspots. We have currently no explanation for this characteristic but we speculate that it may be related to the accessibility of the chromosomal DNA [13,20]. Transposon exclusion by high-density DNA binding proteins was described during the transposition of phage Mu in *Salmonella enterica* serovar Typhimurium [55]. The authors proposed that transcription insulated genes from transposition, which could explain the low level of insertions observed here at the 16S–23S operons. They also showed that a 53 kb region acquired by horizontal transfer was a coldspot and this appears to be the case for other transposable elements as well. The H-NS from *P. aeruginosa* (MvaT and MvaU) which act as global regulatory genes have been shown to bind preferentially to AT-rich regions of the DNA [56]. This would explain the rarity of the insertions into the AT-rich LPS biosynthesis locus, a region which also evolved by horizontal transfer [57].

Very interestingly, the fraction of transposition events that occur inside a copy of a phage genome showed a pattern of distribution indicating that the events took place in phage genomes topologically constrained in a very structured and stable way. The organization of the phage Mu genome, including the prophage, in a stable domain, was previously demonstrated [42,58,59]. We propose in the present work that the domain is organized in four loops structured by the transposome at one end and the strong gyrase site at the other, at least in the Mu-like Ab30 phage. It will be interesting to investigate if this feature is shared among other transposable phages.

This is the first time that such a large amount of replicative insertion events is characterized providing a landscape of phage-host genome interactions. Transposition insertion libraries have been used to qualify genes as essential for bacterial fitness but some authors demonstrated the existence of major biases [60,61]. The present experiment in which bacterial fitness is not a selective factor is an additional illustration of this issue. Indeed there is no opportunity for selection in the process of phage transposition and, in spite of this, we did observe hotspots and coldspots. In a transposition insertion experiment, this might have been wrongly interpreted in terms of fitness of the associated loci.

## 5. Conclusions

This work takes advantage of the deep sequencing technology to investigate the behavior of three transposable phages during replicative transposition at an unprecedented resolution. The mapping of thousands up to millions of transposition sites allowed for the demonstration that within the same bacterial species transposable phages display strikingly different behaviors. This mapping also revealed the existence of hotspots and coldspots for insertion. In addition, investigations of rare insertions into phage genomes provide the first tentative image of the phage chromosomal domain supercoiling into four stable loops in addition to the transposome. The systematic analysis of phage/host junctions in sequencing reads produced as part of phage sequencing should be performed on additional transposable phages to characterize their transposition behavior and decipher the genetic control of these fundamental processes. In particular, the role of the bacterial host’s genetic background will need to be investigated.

## Figures and Tables

**Table 1:** Main characteristics of phages Ab30, 2P1, and HW12.

Features	vB_PaeS_PAO1_Ab30	vB_PaeS_PcyII-10_2P1	vB_PaeS_PAO1_HW12
common name	Ab30	2P1	HW12
source	sewage water	sewage water	clinical isolate
geographic origin	Abidjan, Côte d’Ivoire	Orsay, France	Moscow, Russia
year of isolation	2012	2014	1983 *
genome size	37,205 bp	37,087 bp	37,496 bp
accession number	LN610590	LN907801	LT999987
host strain for phage growth	PA14	PcyII-10	PAO1
sequencing reads accessions	ERR1596849	ERR1596851	ERR2402365
Average phage sequencing coverage	1200×	10,000×	12,000×
publication	[31]	this report	[25]

* Year of recovery of the phage from the clinical *P. aeruginosa* strain provided to V.K. by Professor Bruce Holloway.

**Table 2:** A list of the 14 main Ab30 insertion hotspots (at least one nucleotide position with more than 3000 events).

#	Position PA14 NC_008463 *	Position PAO1 NC_002516 *	CDS Locus Tag **	Distance *Nsi*I $	Ratio Forward $	Distance from Consensus Motif (bp) £	Mismatches £	VNTR §
1	254,808; 254,824	265,008; 265,024	PA14_RS01160	28,587/24,475	41%	56	2	ms211
2	806,635; 806,652	4,720,167; 4,720,184	PA14_RS30080	2414/16,824	58%	58	0	
3	1,569,481; 1,569,485	3,988,644; 3,988,648	PA14_RS07340	68,541/26,332	47%	57	+	
4	1,682,632; 1,682,636	3,858,411; 3,858,415	between PA14_RS07780 and PA14_RS07785	299/18,473	35%	57	2	
5	2,879,877; 2,879,884	2,752,911; 2,752,918	PA14_RS13425	336/4118	35%	57	3	
6	2,938,509; 2,938,513	2,705,279; 2,705,283	PA14_RS13610	4732/32,396	48%	56	0	ms214
7	3,079,036; 3,079,040	2,558,697; 2,558,701	between PA14_RS14100 and PA14_RS14105	1443/34,807	50%	56	0	
8	3,079,172	2,556,658	PA14_RS14105	1579/34,675	47%	60	0	
9	3,325,225; 3,325,229	3,874,426; 3,874,430	between PA14_RS15200 and PA14_RS15205	6907/23,919	61%	57	0	
10	3,555,249; 3,555,253	2,077,078; 2,077,082	PA14_RS16160	495/7668	47%	60	0	
11	5,214,540; 5,214,544	5,050,834; 5,050,838	between PA14_RS23875 and PA14_RS23880	8737/1571	54%	57	+	
12	5,725,984; 5,725,999	5,455,320; 5,455,335	between PA14_RS26245 and PA14_RS26250	44,700/98	80%	54	1	ms223
13	5,732,031; 5,732,035	5,461,367; 5,461,371	PA14_RS26270	5927/9363	51%	59	1	
14	5,743,027; 5,743,031	5,471,620; 5,471,624	between PA14_RS26335 and PA14_RS26340	1622/2499	58%	58	1	ms224

# the major hotspots numbered from 1–14 as shown in Figure 2. * the indicated positions in the PA14 or PAO1 reference genomes are immediately adjacent to at least 3000 right-ends; insertion of the phage DNA is not necessarily elevated at internal positions as illustrated in Figure 3 for hotspot 11; ** the intra or intergenic status of the hotspots is indicated. $ the distance from the hotspots to the nearest flanking *Nsi*I sites is indicated together with the ratio of forward versus reverse integrations of the phage genome. A significant and artefactual bias is observed due to the host-phage junctions enrichment process used, when the hotspot is immediately adjacent to an *Nsi*I site as in hotspots 4, 5, and 12. £ distance in base-pairs between the hotspot and the motif, and number of mismatches between the motif and the 35 bp consensus, as shown in Figure S4 and Figure 4. +, indel in the motif compared to the consensus. §, the Variable Number of Tandem Repeats (VNTR). Some motifs are part of a tandemly repeated element used in genotyping assays [35,36].

## Supplementary Materials

The following are available online at http://www.mdpi.com/1999-4915/10/5/245/s1: Figure S1: the enrichment in Ab30 phage ends; Figure S2: clustering analysis of the phage’s genome sequences including close neighbors; Figure S3: phage 2P1 distribution of transposition sites in host strain genome PcyII-10; Figure S4: detection of a conserved region in the vicinity of the fourteen strongest Ab30 transposition hotspots; Figure S5: Mapping of 25,000 Ab30 transposition sites across PAO1 illustrates the difference with the HW12 behavior and the similarity with 2P1; Figure S6: the correlation of transposition cold-spots with low GC content segments; Figure S7: a candidate for a Strong Gyrase-binding Site in the phage domain; Table S1: coincident insertions, expected and observed in the three phages; Table S2: the association between VNTR loci and Ab30 hotspots; and Table S3: the position of hotspot associated motifs and amount of associated events (maximum and average).
