# Supplementary material for: Transposition Behavior Revealed by High-Resolution Description of Pseudomonas Aeruginosa Saltovirus Integration Sites
Source: Viruses. 2018 May 7;10(5):245. doi: 10.3390/v10050245 (PMC5977238; doi:10.3390/v10050245)
Supplement: Supplementary file 1 [file viruses-10-00245-s001.zip › viruses-296269_supplementary files/TableS2_the association between VNTR loci and Ab30 hotspots.docx]

Table S2. Association between VNTR loci and Ab30 transposition hotspots

| VNTR* | Position in PA14 NC_008463** | Consensus Motif*** | Hotspot # | Size *in vitro*$ |
| --- | --- | --- | --- | --- |
| ms211 | 254 563 – 254 779 | 254 751 | 1 | 2 (258) |
| ms215 | 1 148 525 – 1 148 991 | 1 148 680 & 1 148 810 |  | 2 (507) |
| ms142 | 1 666 988 – 1 667 150 | 1 667 038 |  | 2 (201) |
| ms214 | 2 937 953 – 2 938 564 | 2 938 108 & 2 938 223 &2 938 337 & 2 938 452 | 6 | 5 (656) |
| ms222 | 5 633 069 – 5 633 418 | 5 633 278 |  | 2 (390) |
| ms223 | 5 725 596 – 5 726 009 | 5 676 019 & 5 725 718 & 5 725 823 & 5 725 929 | 12 | 4 (454) |
| ms224 | 5 742 974 – 5 743 447 | 5 742 968 | 14 | 3 (514) |

* Variable Number of Tandem Repeat loci as described in Vu-Thien *et al*., J Clin Microbiol 2007, 45, 3175-3183 and Sobral et al., Eur J Clin Microbiol Infect Dis 2012, 31, 2247-2256.

** Region covered by the VNTR

*** Coordinate of one end of the hotspot associated motif shown in Figure 4

# Associated hotspot as numbered in Figure 2

$ VNTR allele coding convention (and PCR product size in bp) corresponding to repeat copy number rounded up to next integer in PA14 according to Sobral *et al*., Eur J Clin Microbiol Infect Dis 2012, 31, 2247-2256.
