# Supplementary figures and images for: Transposition Behavior Revealed by High-Resolution Description of Pseudomonas Aeruginosa Saltovirus Integration Sites
Source: Viruses. 2018 May 7;10(5):245. doi: 10.3390/v10050245 (PMC5977238; doi:10.3390/v10050245)

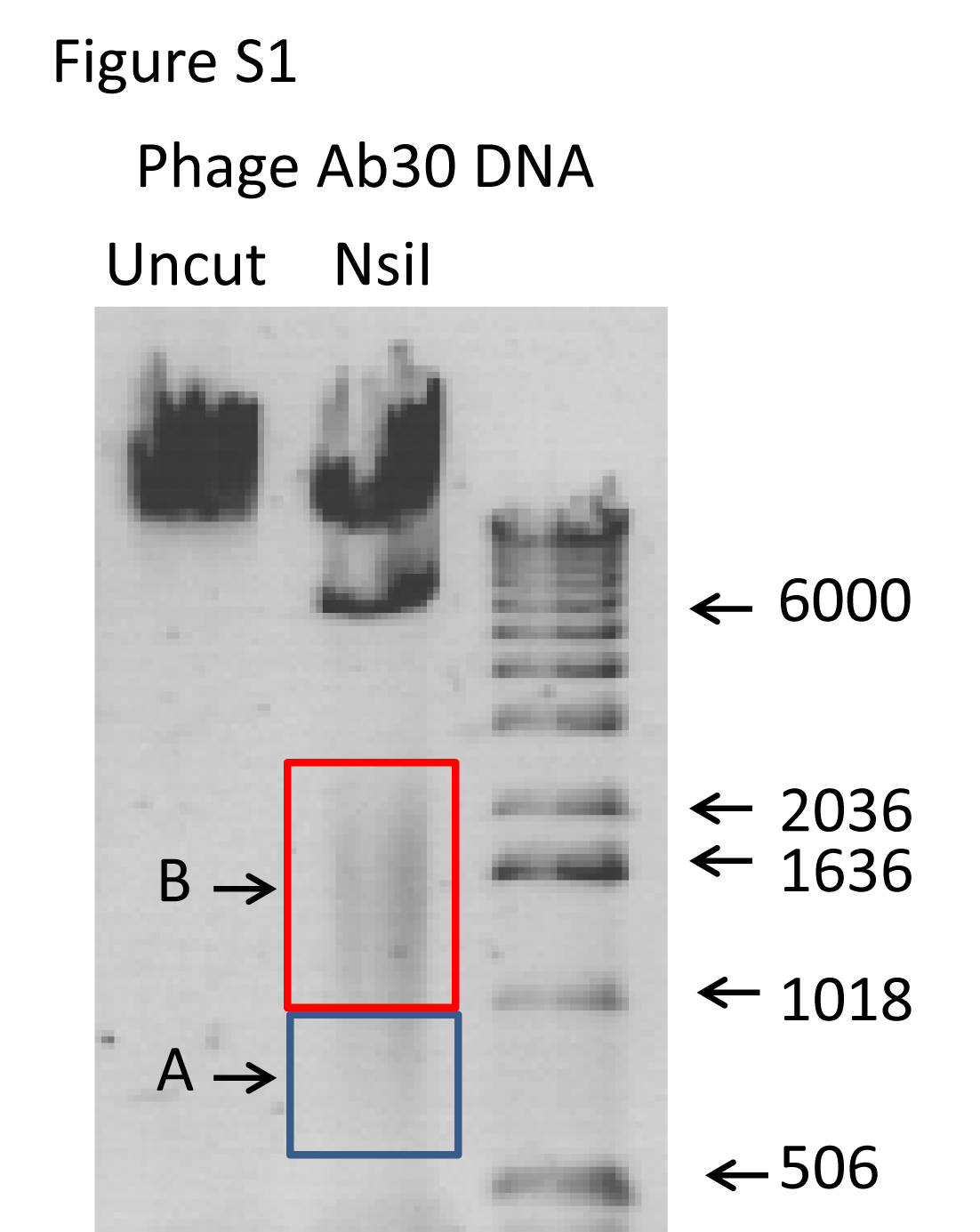

Supplement: Supplementary file 1 [file viruses-10-00245-s001.zip › viruses-296269_supplementary files/FigureS1_the enrichment in Ab30 phage ends.tif]

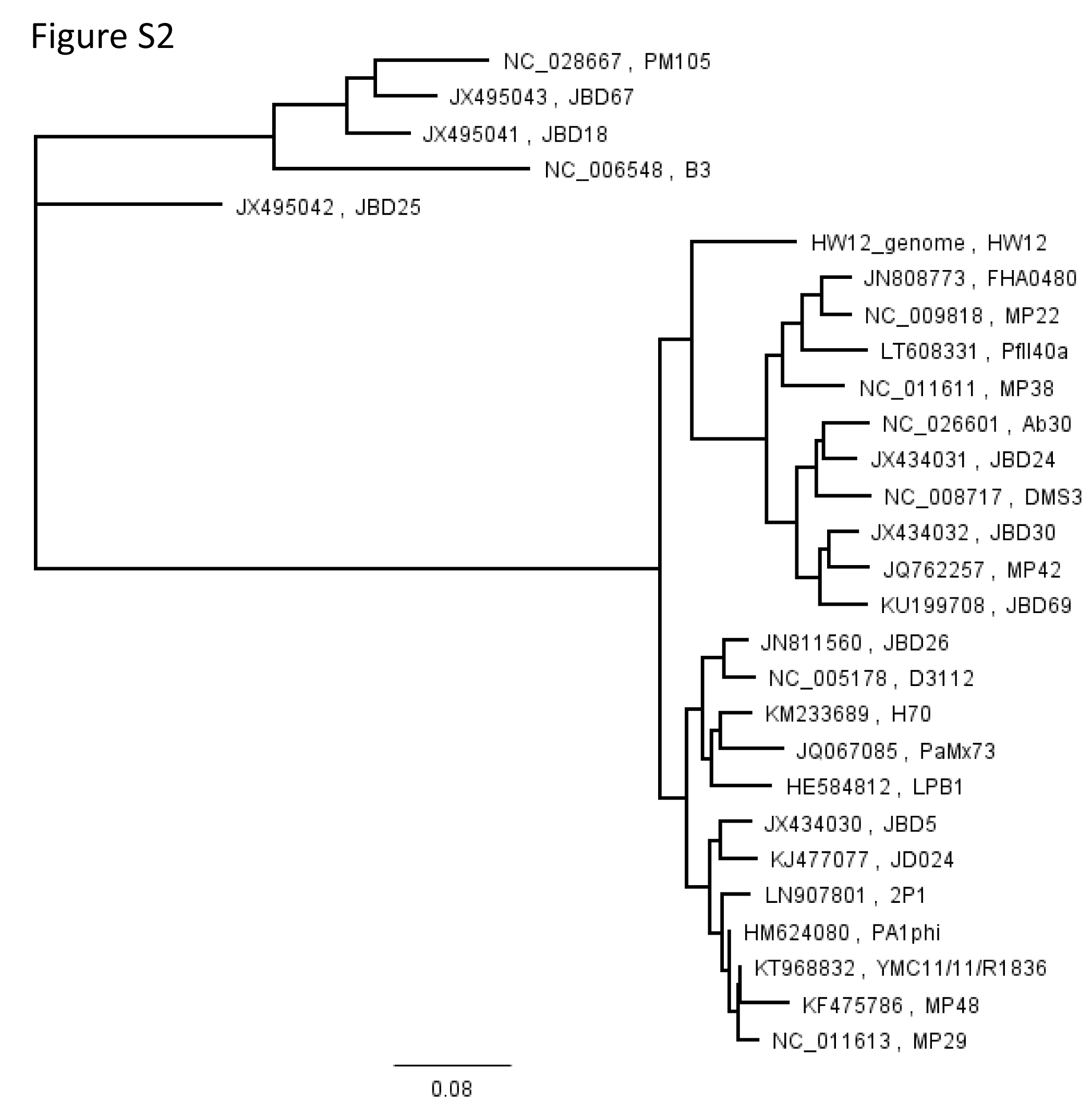

Supplement: Supplementary file 1 [file viruses-10-00245-s001.zip › viruses-296269_supplementary files/FigureS2_clustering analysis of the phage’s genome sequences including close neighbors.tif]

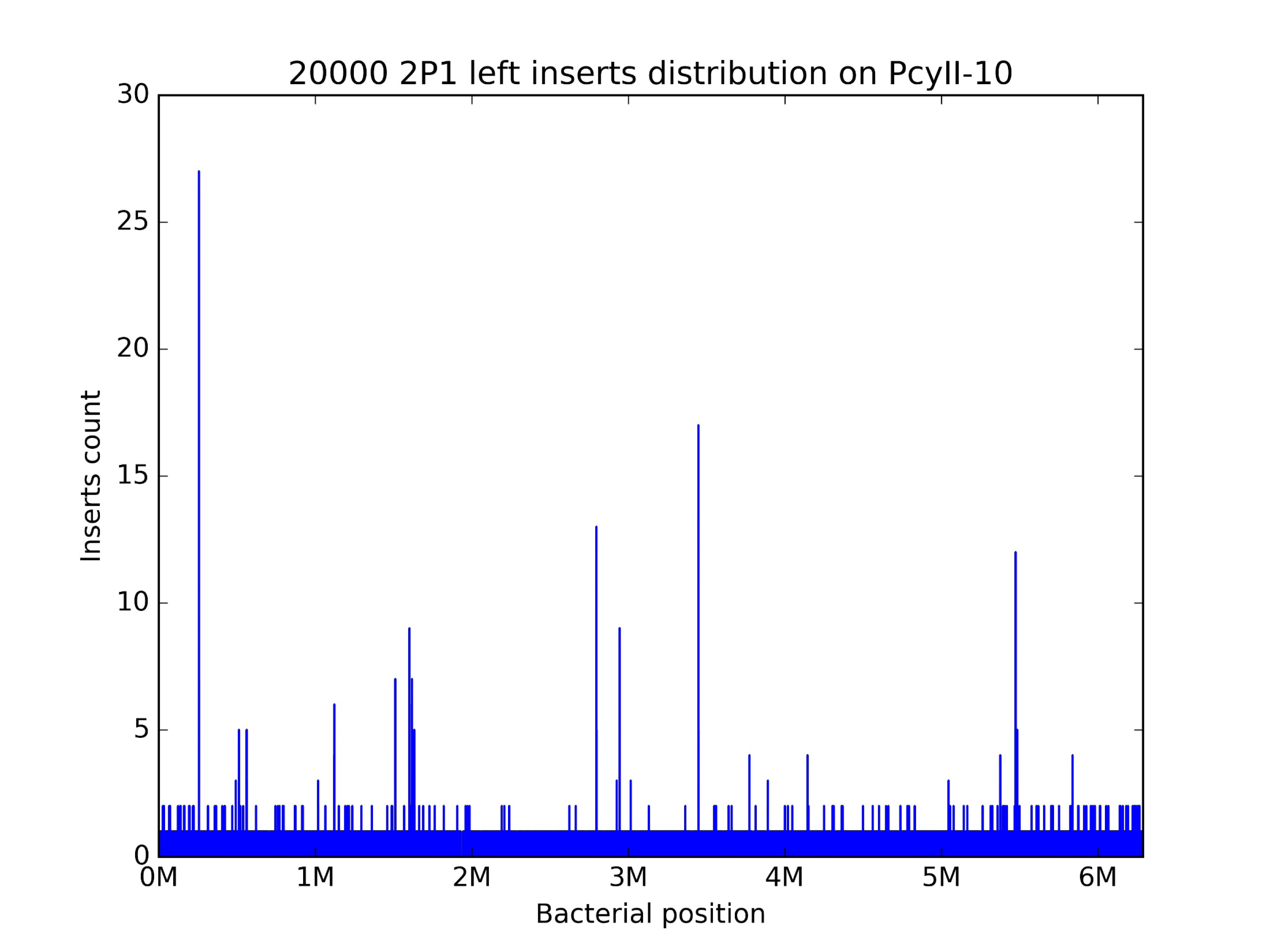

Supplement: Supplementary file 1 [file viruses-10-00245-s001.zip › viruses-296269_supplementary files/FigureS3_phage 2P1 distribution of transposition sites in host strain genome PcyII-10.tif]

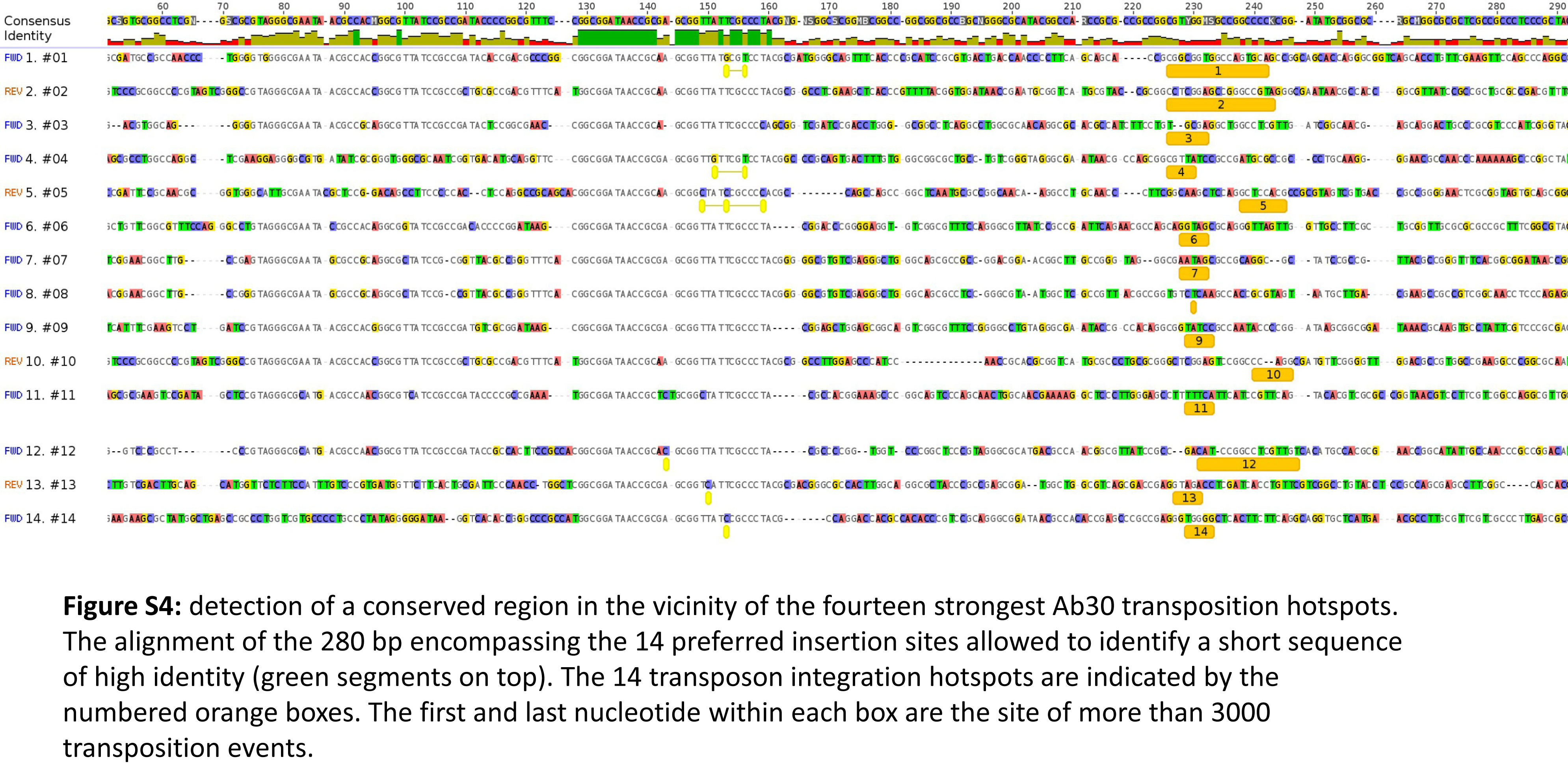

Supplement: Supplementary file 1 [file viruses-10-00245-s001.zip › viruses-296269_supplementary files/FigureS4_detection of a conserved region in the vicinity of the fourteen strongest Ab30 transposition hotspots.tif]

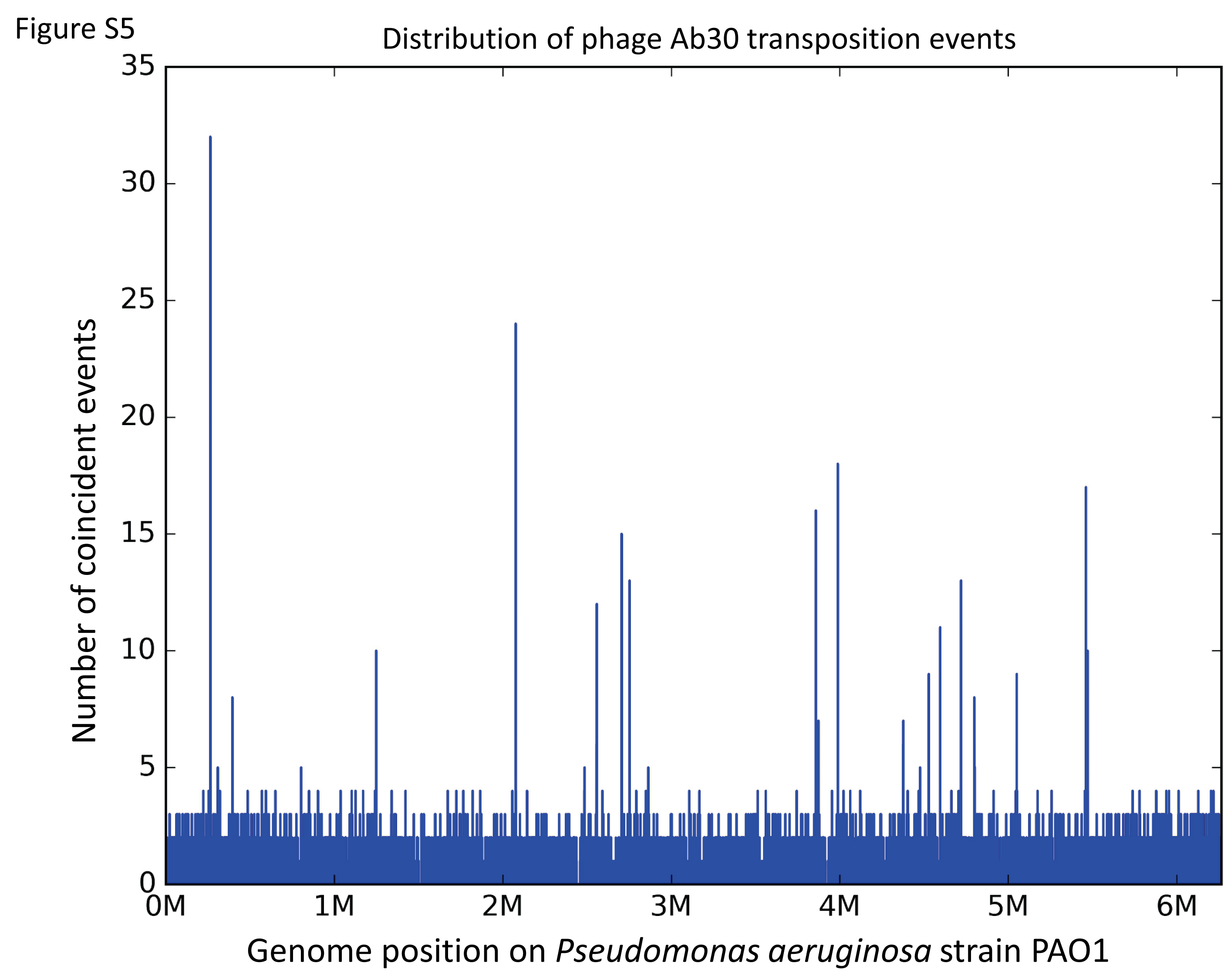

Supplement: Supplementary file 1 [file viruses-10-00245-s001.zip › viruses-296269_supplementary files/FigureS5_Mapping of 25,000 Ab30 transposition sites across PAO1 illustrates the difference with HW12.tif]

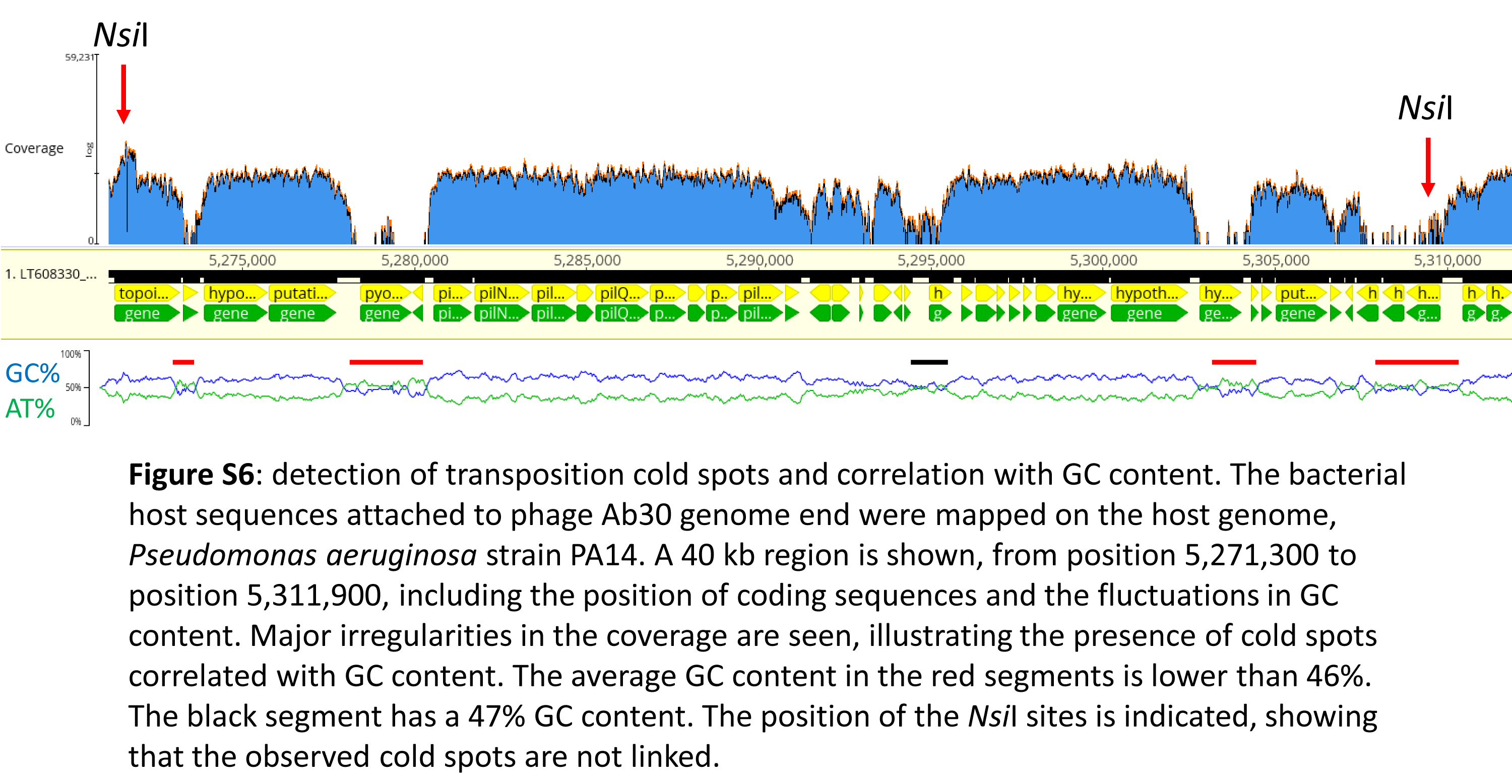

Supplement: Supplementary file 1 [file viruses-10-00245-s001.zip › viruses-296269_supplementary files/FigureS6_the correlation of transposition cold-spots with low GC content segments.tif]

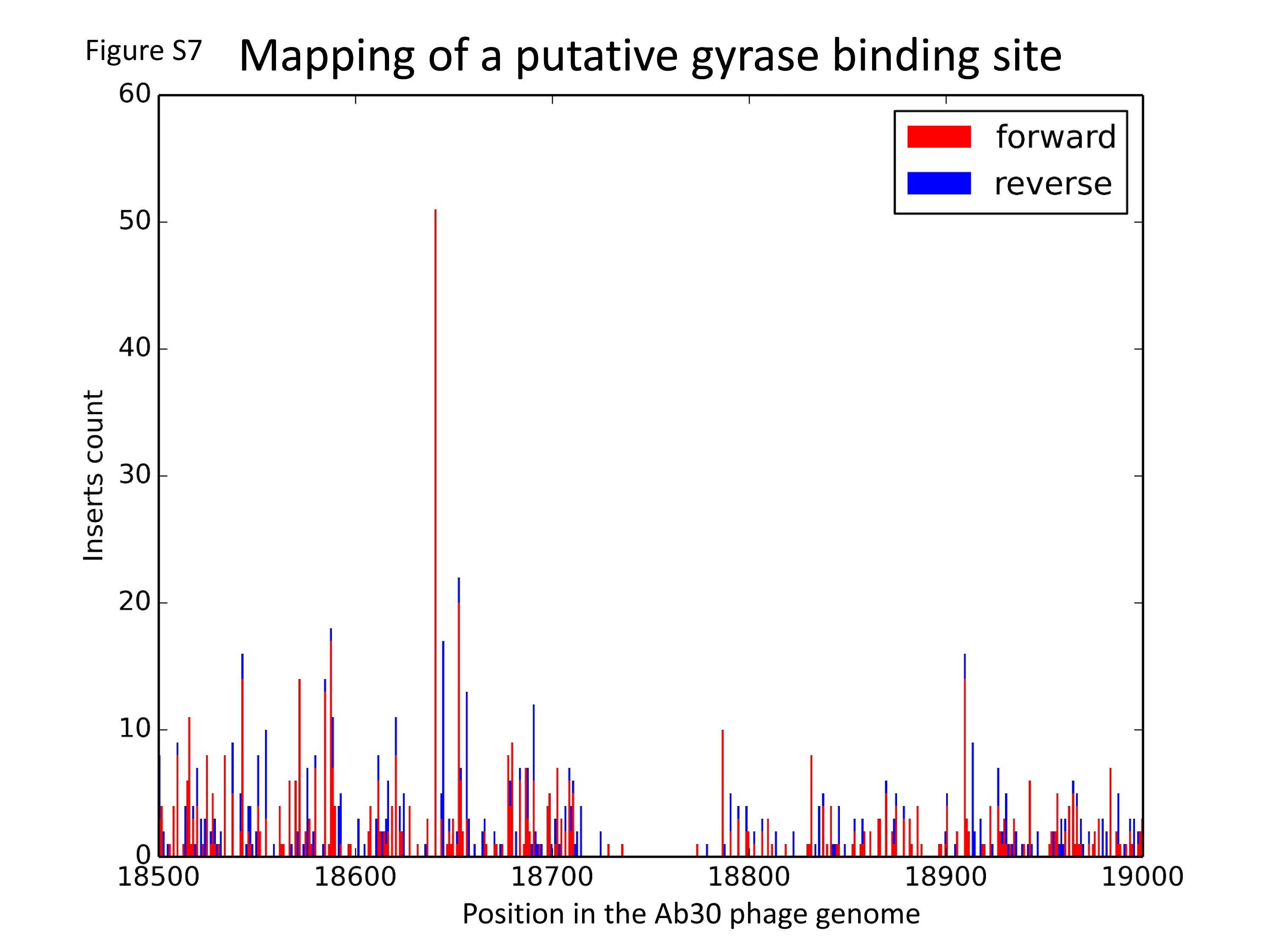

Supplement: Supplementary file 1 [file viruses-10-00245-s001.zip › viruses-296269_supplementary files/FigureS7_a candidate for a Strong Gyrase-binding Site in the phage domain.tif]
